# Image registration and appearance adaptation in non-correspondent image regions for new MS lesions detection

**DOI:** 10.3389/fnins.2022.981523

**Published:** 2022-09-07

**Authors:** Julia Andresen, Hristina Uzunova, Jan Ehrhardt, Timo Kepp, Heinz Handels

**Affiliations:** ^1^Institute of Medical Informatics, University of Lübeck, Lübeck, Germany; ^2^German Research Center for Artificial Intelligence, Lübeck, Germany

**Keywords:** convolutional neural networks, non-correspondences, image registration, shape and appearance adaptation, multiple sclerosis, new lesions

## Abstract

Manual detection of newly formed lesions in multiple sclerosis is an important but tedious and difficult task. Several approaches for automating the detection of new lesions have recently been proposed, but they tend to either overestimate the actual amount of new lesions or to miss many lesions. In this paper, an image registration convolutional neural network (CNN) that adapts the baseline image to the follow-up image by spatial deformations and simulation of new lesions is proposed. Simultaneously, segmentations of new lesions are generated, which are shown to reliably estimate the real new lesion load and to separate stable and progressive patients. Several applications of the proposed network emerge: image registration, detection and segmentation of new lesions, and modeling of new MS lesions. The modeled lesions offer the possibility to investigate the intensity profile of new lesions.

## 1. Introduction

Multiple sclerosis (MS) is a chronic inflammatory disease that progressively destroys the axons in the central nervous system. With an estimated number of more than 2 million affected, MS is the leading cause of neurological disability in young adults (WHO, 2008). The detection and quantification of new MS lesions based on magnetic resonance (MR) imaging is a crucial task in the monitoring of MS, since the presence of new lesions indicates drug inefficacy. The manual segmentation of MS lesions, however, is time-consuming and complex. In a postmortem study (Geurts et al., 2005), only 40% of lesions detected on histopathology were also found on FLAIR MR scans. The detection of new lesions is considered to be an even more challenging task, exhibiting high intra- and inter-rater variance. The automation of (new) MS lesion detection and segmentation has therefore attracted substantial attention recently, e.g., through several public challenges (Commowick et al., 2016, 2021; Carass et al., 2017).

Existing methods for automatic longitudinal examination of MS may be classified into lesion detection and change detection approaches (Lladó et al., 2012). Lesion detection approaches segment all lesions on MR volumes of single time points. For a longitudinal quantification of changes, a subsequent differentiation of static, dynamic and new lesions is needed. Köhler et al. for example use a semi-automatic segmentation approach to mark lesions in individual MR scans. Afterwards, they affinely register all images to a reference scan and finally distinguish between stable, dynamic and new lesions based on the intersection of lesion masks from all time points (Köhler et al., 2019).

Change-detection approaches on the other hand directly use both images from subsequent time points to detect changes between baseline and follow-up. These approaches can be subclassified into intensity- and deformation-based approaches (Salem et al., 2020). Intensity-based approaches compare pre-registered scans of subsequent time points on a voxel-by-voxel basis to segment new lesions, e.g., Moraal et al. (2010), Ganiler et al. (2014), and Battaglini et al. (2014); Jain et al. (2016); Fartaria et al. (2019). Deformation-based approaches, however, use non-rigid image registration and analyze the resulting deformation fields to find new or evolving lesions (Rey et al., 2002; Cabezas et al., 2016). Works combining ideas from intensity- and deformation-based approaches show improved performance compared to using intensity-based solutions alone (Cabezas et al., 2016; Salem et al., 2018).

The majority of recent methods for new MS lesion segmentation are based on deep learning (Krüger et al., 2020a; McKinley et al., 2020; Salem et al., 2020; Combès et al., 2021). A trend reflected in the submissions to the MICCAI 2021—Longitudinal Multiple Sclerosis Lesion Segmentation (MSSEG-2). Challenge (Commowick et al., 2021), most of which perform image registration as a pre-processing step and subsequently use a 2D or 3D U-Net-like architecture to segment new lesions. Especially promising segmentation results are achieved by Dalbis et al. (2021) and Zhang et al. (2021) that both use a 2.5D approach with image slices of all three directions as network input.

Salem et al. (2020) propose a fully convolutional network (FCN) that consists of four registration blocks followed by a segmentation block. Each registration block registers the baseline scan of a certain modality (T1, T2, PD, and FLAIR) to the respective follow-up scan. The resulting deformation fields are then fed to the segmentation part of the network (Salem et al., 2020). For the MSSEG-2 challenge, the authors adapt their approach to work with FLAIR images only.

Using image registration as a pre-processing step to lesion load change or new lesions detection may cause underestimation of changes, since not only geometrical distortions but also changes of interest are erroneously eliminated by the registration step. Joint image registration and non-correspondence estimation may overcome this problem (Dufresne et al., 2020). Classic, i.e., iterative approaches that estimate non-correspondences during the registration process can be found in (Ou et al., 2011; Chen et al., 2015; Dufresne et al., 2020; Krüger et al., 2020b). Ou et al. (2011) estimate the matching uniqueness between voxel pairs to weigh the image distance measure during the registration process. A similar approach is followed by Krüger et al. (2020b) who use probabilistic correspondences between sparse image representations to define the weight map. In Chen et al. (2015) and Dufresne et al. (2020), a segmentation mask of non-corresponding regions is generated during the registration process. This segmentation is used to mask out the image distance measure in non-corresponding image regions. Together with regularization of the segmentation, non-corresponding regions are thus found as outliers in the image distance and segmented directly. Following this approach, we propose in Andresen et al. (2022) what is, to the best of our knowledge, the first method that tackles joint image registration and non-correspondence segmentation with deep learning. For the MSSEG-2 challenge, we use this approach to register baseline and follow-up images of MS patients while simultaneously segmenting non-corresponding regions. The non-correspondence segmentation is then refined with a second FCN, resulting in a final segmentation of new MS lesions (Andresen et al., 2021).

While all these approaches handle non-correspondences by weighing them down during the registration process, other methods for image registration with non-correspondences directly model both spatial and intensity differences between images to make them look alike (Trouvé and Younes, 2005; Rekik et al., 2015; Wilms et al., 2017; Bône et al., 2020). Uzunova et al. propose the joint shape and appearance autoencoder (SAAE) that reconstructs images from a global template using spatial deformations and intensity transformations (Uzunova et al., 2021). This allows the reconstruction of different modalities within the same framework. To assure a proper disentanglement of shape and appearance, guided filtering (He et al., 2013) is used such that the appearance offsets do not change the shape of the template.

Inspired by Uzunova et al. (2021), we now extend our image registration CNN for new MS lesions detection (Andresen et al., 2021) to ANCR-Net (appearance adaptation in non-correspondent regions and image registration network). ANCR-Net not only spatially deforms the baseline image, but also changes its appearance in non-corresponding image areas to match the follow-up. The spatial displacement accounts for general misalignments between the baseline and the follow-up images, as well as for old lesions changing shapes and sizes. The intensity transformations, however, are not applied to the entire baseline images but only in non-corresponding areas, which allows us to directly model newly appearing MS lesions. Different from Andresen et al. (2021), we use only one CNN whose segmentation branch is trained in a supervised manner. The trained network offers several applications for MS lesion analysis: 1) detection and segmentation of new lesions, 2) registration of baseline to follow-up images and 3) modeling the appearance of new lesions.

## 2. Materials and methods

### 2.1. Training objective

As described in Andresen et al. (2022), CNN-based image registration of baseline image B:Ω → ℝ and follow-up image F:Ω → ℝ with simultaneous non-correspondence segmentation can be formulated with the following training objective (Andresen et al., 2022).


(1) $$\begin{array}{l} {ℒ}_{\text{NCR}}=(1-N)\cdot \text{D }(\text{F},\text{B}\circ \;\varphi \;)+\alpha {\text{R}}_{\varphi }+\beta {\text{R}}_{N}, \end{array}$$

with image distance measure D and regularizers R_φ_ and R_*N*_. The diffeomorphic deformation field φ:ℝ → ℝ^3^, with φ = exp(*v*) and the segmentation of non-correspondences *N*:Ω → [0, 1] are both network outputs. The regularizers ${\text{R}}_{\varphi }=||\nabla v|{|}_{2}^{2}$ and ${\text{R}}_{N}=\underset{x\in \Omega }{\sum }N+\gamma \text{tanh}(||\nabla N|{|}_{2})$ enforce smoothness of the velocity field *v* and small, regularly bordered segmentations *N*. The image distance measure D is evaluated only in corresponding image regions, while non-corresponding areas with large image distance are masked out. Based on outlier detection in the image distance measure, the network is able to simultaneously segment non-correspondences and to spatially align baseline and follow-up images.

Taking ideas from Uzunova et al. (2021), we now want to model new MS lesions as appearance offsets between baseline and follow-up images in non-corresponding image regions. This results in the new training objective


(2) $$\begin{array}{l} ℒ=\text{D}(\text{F},(\text{B}+N\cdot A)\circ \varphi )+\alpha {\text{R}}_{\varphi }+\beta {ℒ}_{\text{Dice}}(N\circ \varphi ,\text{S}). \end{array}$$

Appearance offsets *A*:Ω → ℝ are masked with the non-correspondence segmentation *N* and added to the baseline image. The appearance adapted baseline is then spatially deformed to match the follow-up image. Normalized cross-correlation is used as an image distance measure. The regularizer R_φ_ is defined as in Eq. (1). Other than our previous approach, we now use the Dice loss between the network's non-correspondence segmentation *N* and the ground truth segmentation S, making the regularization of *N* obsolete.

The intuition behind this method is that only in the regions of new lesions, strong intensity changes are to be expected between the baseline and the follow-up. Thus, intensity transformations are only applied in the non-corresponding image regions in order to directly model the newly appearing MS lesions. The spatial displacement φ in turn accounts for old lesions changing shapes and sizes as well as for general misalignments between the baseline and the follow-up images, but not for newly appearing lesions.

### 2.2. Network architecture

**Figure 1:**
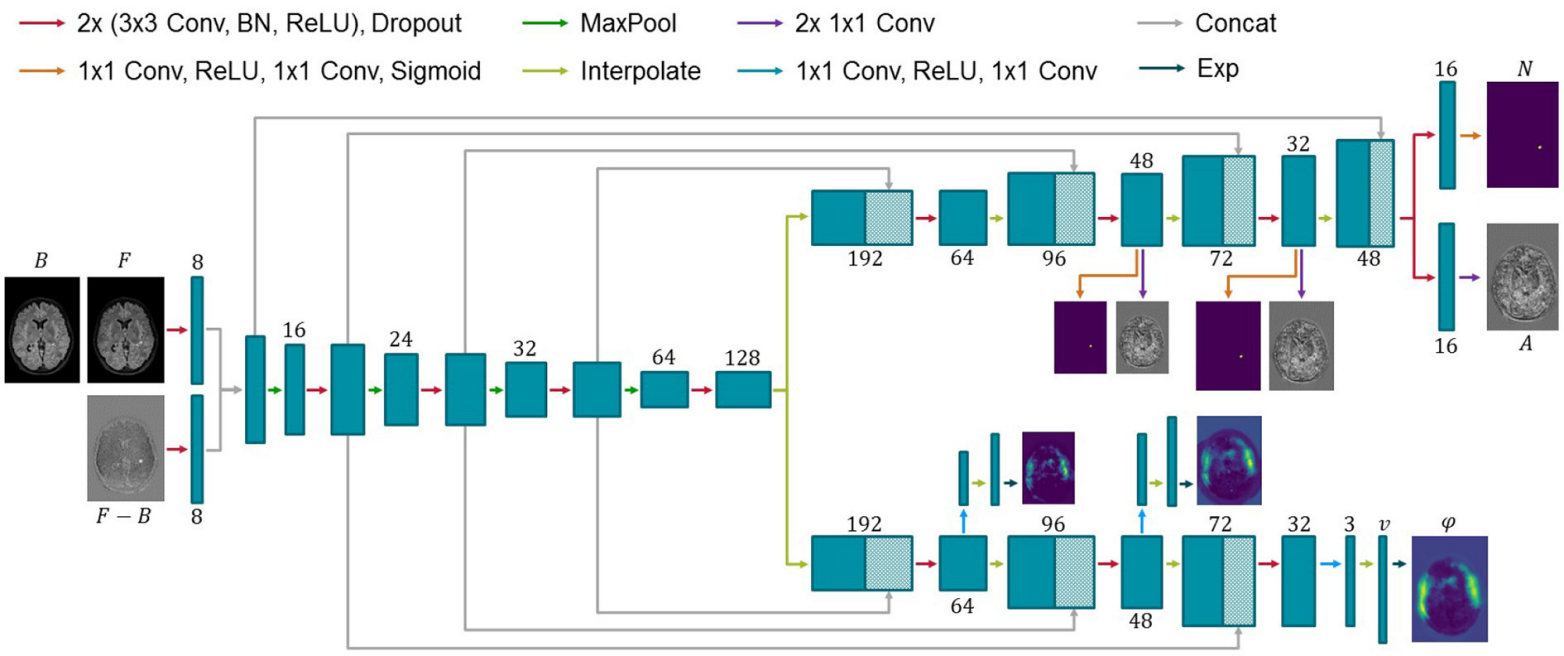
Architecture of the proposed network for new MS lesions modeling. Input to the network are baseline and follow-up MR images and their subtraction image. Two decoders generate a diffeomorphic deformation field φ for registration of baseline to follow-up and a segmentation *N* of new lesions together with appearance offsets *A*. Numbers below or above the blue boxes indicate the number of feature maps. Since the max pooling does not change the number of features, we omit the numbers before max pooling for visualization purposes.

Consistent with previous works, the proposed ANCR-Net consists of one encoder and two separate decoders whose exact architecture is shown in Figure 1. The encoder starts with two separate convolutional blocks that process input MR images and their subtraction image. The resulting feature maps are concatenated and passed through multiple max pooling and convolution operations, analogously to the U-Net (Ronneberger et al., 2015). Another common feature to the U-Net is that our network also has decoders connected to the encoder *via* skip connections. The first decoder outputs the diffeomorphic deformation φ and the other generates non-correspondence and appearance offset maps *N* and *A*. Outputs are generated on three levels of resolution to provide deep supervision on both branches (Hering et al., 2019; Andresen et al., 2022). The loss function is determined at all three levels of resolution and a weighted sum is calculated to give a final loss for backpropagation. The weighting factors are chosen to be 0.7, 0.2 and 0.1 for each level, respectively, giving the finest resolution level the highest weight. Input to the network are five stacked axial slices sampled to an isotropic resolution and image size of 368 × 512 pixels. To generate segmentation results for the entire image volume, we iterate slice-wise through the volume and keep the segmentation of the central slice of the stacked input patches.

### 2.3. Network training

For network training, we use the MSSEG-2 challenge dataset. It consists of 40 whole-head FLAIR MR image pairs. Baseline and follow-up images have been rigidly pre-aligned for each patient. New MS lesions—if present—were manually segmented in the pre-aligned images by four medical experts and combined to one ground truth label of new lesions, which are used for network training.

New MS lesions are rare and mostly small, resulting in lesions being severely underrepresented in the data. To account for the class imbalance problem, we pre-train the network by inserting simulated lesions into the images that do not have real new lesions and deforming them with random elastic deformations. The network is then trained in a supervised manner using Dice loss and mean squared error between predicted and ground truth deformations as loss function. For lesion simulation, we generate a mask indicating candidate locations of lesions as follows. First, brain extraction is performed on both time points separately and the union of the brain masks is defined as the final brain mask. Second, baseline and follow-up images are normalized to values between 0 and 1 and thresholded above 0.1 to exclude the ventricles from the final mask. The brain mask is then multiplied with the thresholded MR images. As the simulated lesions should not protrude beyond the edge of the brain, the mask is subsequently shrunk using morphological erosion.

Artificial lesions are inserted on the fly during pre-training by first selecting a random number of new lesions (minimum one and maximum five) and randomly selecting locations from the candidate locations extracted before. At each selected location, we simulate a new lesion as a Gaussian ellipsoid whose values are added to the image intensities.

After lesion insertion, a random elastic deformation is applied to the image which then serves as fixed image whereas the original image is used as moving image. In addition, the following augmentation techniques are randomly applied to the moving and reference images during both the pre- and the final network training:

- Gaussian noise (inside brain region only)
- Rotation (±5°, performed on both images)
- Shift (±3 pixels in the axial plane, performed on both images)
- Brightness change (inside brain region only)
- Brightness gradient (inside brain region only)
- Adaptive histogram equalization

Pre-training is performed for 200 epochs, Adam optimization and a learning rate of 1e^−4^ that is decayed every 20th epoch with a factor of 0.8. After pre-training, ANCR-Net is trained with the loss function (2) using only image patches containing new lesions in the manual ground truth. Each of these patches is passed twice to the network, once with the original orientation and once flipped horizontally. Training is again performed with Adam optimization, exponentially decaying learning rate starting from 1e^−4^ and run for 400 epochs to assure full convergence. All code is made publicly available at https://github.com/juliaandresen/ANCRNet.git.

## 3. Experiments and results

The proposed method is validated on the test dataset of the MSSEG-2 challenge, consisting of 60 FLAIR MR image pairs. In our observations, the ground truth segmentation for one patient in the test data (ID 12) is not correct, thus we discard patient 12 from the test set and report results for the remaining 59 patients. For all experiments, we perform five-fold cross-validation on the training data, splitting the dataset into 32 training and 8 validation images per fold. The networks are ensembled and segmentations combined by majority vote. Each lesion in the resulting segmentations that is smaller than 3mm^3^ in volume is discarded. All metrics reported for new lesions detection and segmentation compare the manual consensus ground truth with the non-correspondence segmentations *N*. The non-correspondence segmentations are multiplied with brain masks generated by the default pre-processing pipeline^1^ before metrics calculation.

### 3.1. New lesions detection

New lesions detection performance is measured with several metrics. First, we report lesion sensitivity Sens_L_, the proportion of detected new lesions in the ground truth. The lesion positive predictive value PPV_L_ gives the proportion of true positive lesions out of all lesions segmented by the network. Finally, the F_1_-Score combines Sens_L_ and PPV_L_ as


(3) $$\begin{array}{l} F_{1}=\frac{2\cdot \text{Sen}{\text{s}}_{\text{L}}\cdot \text{PP}{\text{V}}_{\text{L}}}{\text{Sen}{\text{s}}_{\text{L}}+\text{PP}{\text{V}}_{\text{L}}}. \end{array}$$

These metrics are not suitable for images that do not contain lesions in the ground truth. For these cases, we report average number and volume of erroneously detected lesions. We additionally give the proportion Det_p_ of patients correctly identified as progressing, i.e., at least one ground truth lesion is detected. For patients without new lesions we report Det_s_, the proportion of patients correctly identified as stable, i.e., no segmentation is generated for these patients.

Results are summarized in Table 1 both for images with and without ground truth lesions. For comparison, we report the average performance of the four medical experts who segmented the MSSEG-2 challenge data and of the three teams achieving best results in the four metrics considered at the challenge: MedICL (Zhang et al., 2021) achieving the highest Dice score, Mediaire-B (Dalbis et al., 2021) achieving the best F_1_-Score and LYLE (Ashtari et al., 2021) who performed best for number and volume of erroneously detected lesions. The results per patient can be found in the Supplementary material.

**Table 1:** New lesion detection results for images with and without new lesions in ground truth.

**Model**	**Data**	**With new lesions**				**Without new lesions**		
		**F_1_**	**Sens_L_**	**PPV_L_**	**Det_p_**	**Number**	**Volume**	**Det_s_**
Experts	Validation	0.732	0.697	0.772	0.871	0.000	0.000	1.000
Ours, w/o PT	Validation	0.591	0.634	0.624	0.828	0.545	6.959	0.636
Ours	Validation	0.622	0.666	0.623	0.862	0.455	6.948	0.636
Experts	Test	0.635	0.609	0.663	0.815	0.045	1.514	0.955
MedICL	Test	0.516	**0.760**	0.465	**0.871**	0.536	12.713	0.643
Mediaire-B	Test	0.559	*0.707*	0.507	0.806	0.536	29.235	0.643
LYLE	Test	0.455	0.431	0.522	0.742	**0.036**	**0.470**	**0.964**
Ours, w/o PT	Test	*0.566*	0.623	**0.612**	*0.839*	0.250	4.443	0.750
Ours	Test	**0.582**	0.633	*0.582*	0.806	*0.107*	*2.039*	*0.893*

Reported are average F_1_ score, lesion sensitivity and positive predictive lesion value for images containing new lesions. For stable patients, the average number of erroneously detected lesions and their volume are reported. The results are given for the medical experts who generated the manual ground truth data as well as for our proposed method with and without pre-training (PT) and compared to the three pipelines that performed best in the MSSEG-2 challenge (Ashtari et al., 2021; Dalbis et al., 2021; Zhang et al., 2021). Best results are given in bold font and second best in italics. No method manages to significantly outperform all other methods (according to a Wilcoxon signed rank test with significance level 0.05).

The PPV_L_ results show that most automated methods, including ours, tend to overestimate the number of new MS lesions and generate quite a lot of false positives. This is particularly true when the proposed pre-training is not used. In return, they are able to reliably detect real new lesions, even exceeding the average detection rate of medical experts. Despite the high proportion of false positives on the images with new MS lesions, ANCR-Net manages to correctly identify 89.3% of the 28 patients without a ground truth lesion in the test set as stable. At the same time, an average of 63.3% of ground truth lesions are correctly identified by our network. For 25 out of the 31 patients in the test set, our CNN manages to correctly detect at least one ground truth lesion. Considering not only correctly detected new lesions but all generated lesions, ANCR-Net identifies 29 patients as progressing. While the competitive methods achieve high detection rates either for stable or progressive patients, our method is the only one capable of reliably detecting new lesions and keeping the number of false positives low in stable patients, thus properly separating stable and progressing patients. In addition, our network also reliably estimates the real number of new lesions, with a mean error of only 1.322 lesions.

**Figure 2:**
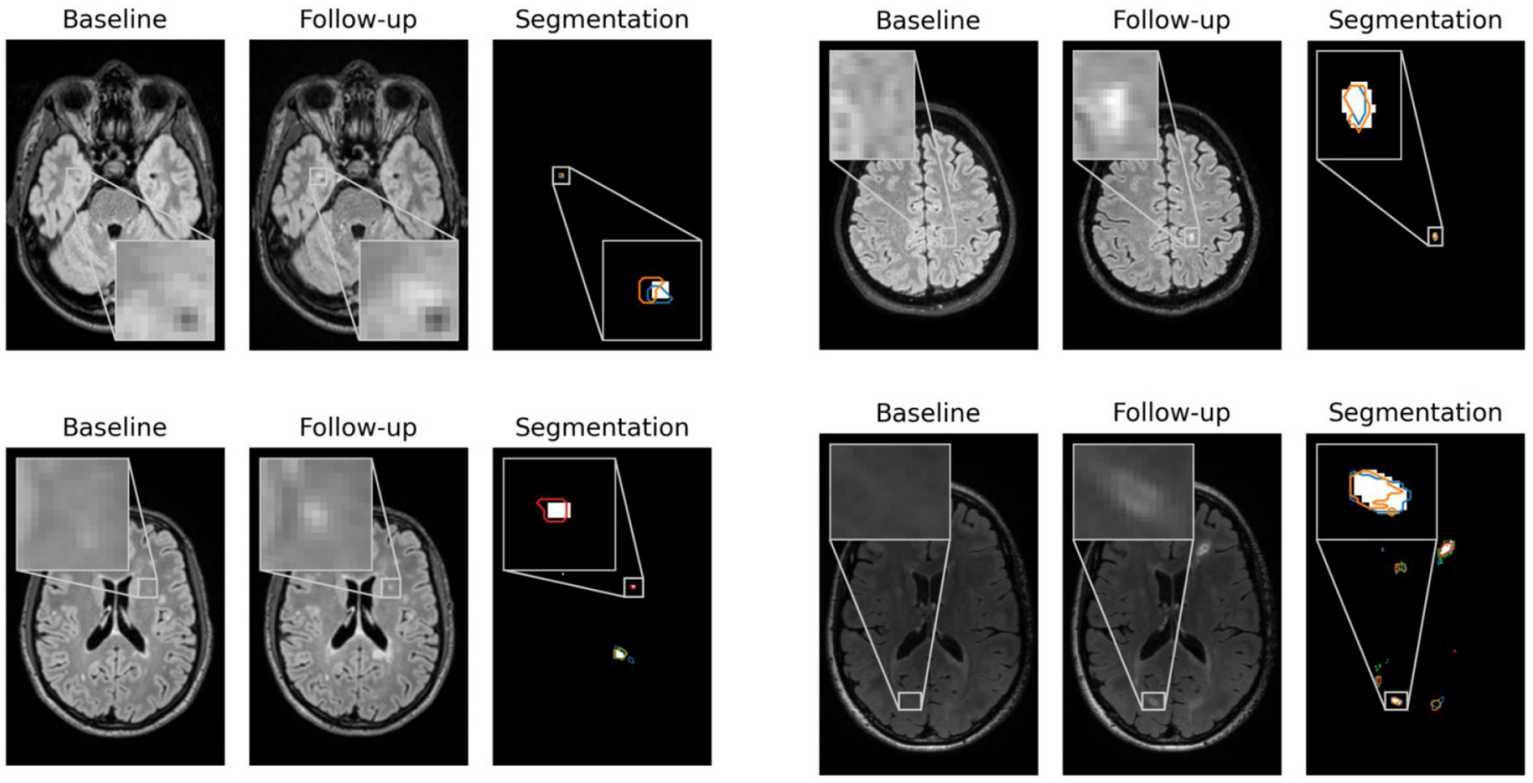
False positives generated by our CNN for patients 1, 23, 62, and 66 that were controversial among the experts. For each patient, baseline, follow-up and the segmentation of new lesions as generated by our network are shown. Segmentation contours of expert 1, 2, 3, and 4 are overlaid in blue, orange, green and red, respectively.

In Figure 2, contentious new lesions not included in the ground truth but segmented by at least one of the four experts and also by our proposed network are shown. The figure highlights the difficulty of the new lesions detection problem that is further aggravated by the changing size and shape of lesions. Automatic methods for new lesion detection inherently suffer from these difficulties, leading to the observed high proportion of false positives.

### 3.2. Segmentation of new lesions

To measure lesion segmentation performance, average Dice score, surface distance and Hausdorff distance are considered. Results are reported in Table 2 and again compared to experts' performance and best performing challenge submissions. Segmentation performances overall are quite low, which is reflected both in the Dice score and in the surface-based metrics. The average surface distance is comparable for almost all automatic methods with a value of just over 9mm. Only LYLE achieves a mean surface distance of 7.209 mm. The results for the Hausdorff distance vary more. Here, too, LYLE performs best with 38.883 mm. Our methods achieves the second lowest value of 42.618 mm.

**Table 2:** New lesion segmentation results for medical experts, our proposed method with and without pre-training (PT) as well as the three approaches performing best in the MSSEG-2 challenge (Ashtari et al., 2021; Dalbis et al., 2021; Zhang et al., 2021).

**Model**	**Data**	**Dice**	**SD**	**HD**
Experts	Validation	0.663	4.013	29.885
Ours, w/o PT	Validation	0.512	7.231^*^	41.502^*^
Ours	Validation	0.502	7.753^*^	37.688^*^
Experts	Test	0.573	6.211	32.639
MedICL	Test	**0.523**	9.352	61.835
Mediaire-B	Test	0.451	9.010^*^	44.866^*^
LYLE	Test	0.422	**7.209** ^*^	**38.883** ^*^
Ours, w/o PT	Test	0.463	12.335	48.167
Ours	Test	*0.470*	9.053^*^	42.618^*^

Reported are average Dice score, surface distance (SD) and Hausdorff distance (HD). Results marked with ^*^ are averaged over non-empty predicted segmentations only. For Mediaire-B two patients and for LYLE five patients are excluded from the distance calculation. With ANCR-Net, two patients from the validation data and two patients from the test data for the version trained with pre-training are excluded from the calculation. Significantly best results are presented in bold font.

Considering Dice score, the best performing method (MedICL) achieves a value of 0.523. Our method scores second with 0.470. Even the experts only achieve an average Dice score of 0.573. This highlights the difficulty of the MS lesion segmentation task. Lesion borders often appear blurred, making their exact delineation difficult. Still, Dice scores do not take into account separate lesions, but only measure the overlap of all segmented pixels. We therefore also compute Dice scores for the test data on lesion-level and report scores averaged over 1) all lesions in ground truth and 2) all detected ground truth lesions. Lesion-wise Dice scores are even lower than the results in Table 2 with 0.412 for our method and 0.558 for the experts when averaging is performed over all ground truth lesions. For detected ground truth lesions, the average lesion-wise Dice score is 0.631, showing that lesion delineation works well in the case of identified lesions, but the gap to experts is still large (experts' average 0.817).

Finally, factors influencing the detection and segmentation quality of ANCR-Net are analyzed. For each lesion in the manual ground truth, volume, convexity, contrast to surrounding tissue and contrast to the baseline image are considered. For lesion volume, the cube root of the volume is used as a very rough estimate of lesion diameter. As described in Lian et al. (2012), the convexity is calculated as the quotient of the lesion volume and the volume of the convex hull of this lesion. To calculate the contrast to the surrounding tissue, we determine the mean intensities within the lesion and in a small area around the lesion (found by binary dilation of the lesion segmentation with a spherical structuring element). The contrast is then calculated as the difference in mean intensity divided by the average of the two mean intensities (Nabavizadeh et al., 2019). The contrast to the baseline image is determined analogously using the mean intensities within the lesion area in baseline and followup images.

**Figure 3:**
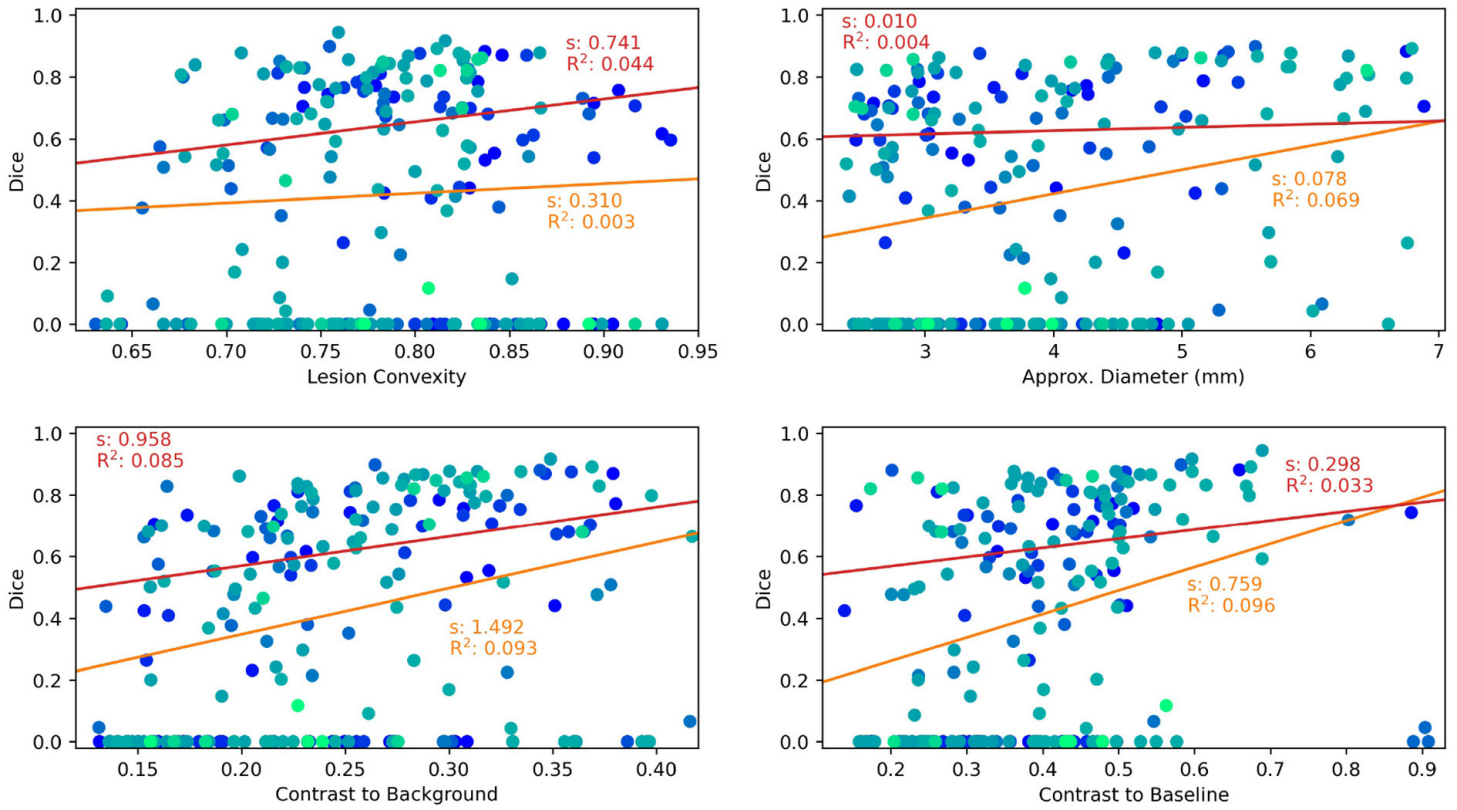
Lesion characteristics influencing the lesion detection and segmentation performance of ANCR-Net. Different colors represent different patients. The results of a linear regression measuring the influence of the respective lesion characteristic on Dice score are shown. Red lines show the results using only those lesions detected by ANCR-Net, whereas orange lines show the results considering all lesions. For each regression line, the slope s and the R^2^ value are given.

Results are shown as scatter plots in Figure 3 where each point represents a ground truth lesion. It can be seen that lesion convexity does not seem to strongly influence the lesion detection performance. The pre-training on artificial lesions with an elliptical shape does not result in better detection of lesions with such a shape (as measured by convexity). The other considered metrics, however, have a greater impact on the detection performance of ANCR-Net. Larger lesions are detected with higher accuracy. Likewise, lesions that show a strong contrast to the background and especially to the baseline image are detected better than lesions with low contrast.

To analyze the influence of the considered lesion characteristics on the segmentation performance of ANCR-Net, linear regression is performed. For each lesion characteristic, we remove outliers biasing the regression results by discarding those lesions whose characteristic is smaller/larger than the 5%-/95% percentile of the respective characteristic. Also, we perform the regression once for all the remaining lesions and once for only those lesions that are detected by ANCR-Net. Each of the considered metrics shows a small positive correlation with Dice-score. A comparison of the regression results for all lesions and only for detected lesions shows again that the lesion volume and the contrast to the baseline image strongly influence the ANCR-Net detection rate. Interestingly, none of the metrics seem to have a very strong impact on segmentation performance when only looking at the detected lesions (red lines in Figure 3). Solely the contrast to the surrounding tissue gives a significant influence on the quality of the segmentation, with an R^2^ of 0.085. Overall, lesion size and contrast to the baseline image are crucial for the detection of the lesions, but less so for their precise delineation, while contrast to the surrounding tissue is more critical for good segmentation.

### 3.3. Modeling of new lesions

**Figure 4:**
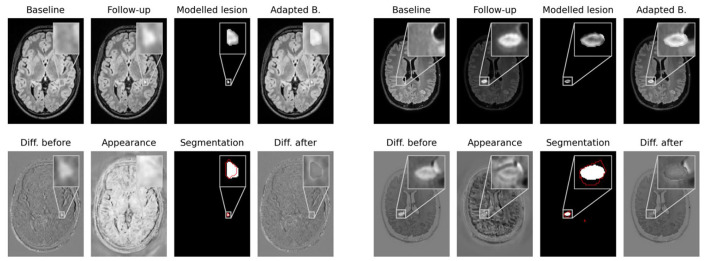
Modeling of new lesions. The appearance map is masked with the new lesions segmentation and added to the spatially deformed baseline image. The upper row shows baseline and follow-up images, the masked appearance map, deformed and appearance adapted baseline. In the lower row the difference image between follow-up and baseline, the appearance map, the segmentation of our CNN and the difference image between follow-up and adapted baseline are shown. The ground truth lesion segmentation is overlaid in red onto the network's segmentation.

Network outputs allow to not only spatially align baseline and follow-up, but also to model the appearance of newly formed lesions. To do so, the appearance offset map masked with the segmentation output is added to the baseline image and the adapted baseline is spatially deformed to match the follow-up image. In Figure 4 some exemplary results are shown for image registration and appearance adaptation between baseline and follow-up using new lesion modeling. For more examples refer to the Supplementary material.

The figure shows that the deformed and appearance adapted baseline images resemble the follow-up images well. The modeled lesions do not overcompensate the overall intensity difference between baseline and follow-up images. Instead, the difference images show similar values inside and outside new lesions. The modeled lesions thus fit the intensity distribution of the baseline image. Investigating the modeled lesions, it can be seen that, even though MS lesions appear primarily as bright spots in FLAIR MR images, some of them still exhibit an irregular intensity profile. These irregular intensity profiles can be seen particularly well in the masked appearance maps (upper row in third and seventh columns in Figure 4), which might be used to analyze the morphology of newly forming MS lesions.

## 4. Discussion

We presented ANCR-Net, a CNN for the adaptation of baseline FLAIR MR images from MS patients to the respective follow-up images. Spatial deformations are applied to align baseline and follow-up structures, and new lesions are simulated in non-corresponding image areas. The trained network gives three outputs, namely a diffeomorphic deformation field to spatially align baseline and follow-up, a segmentation of new lesions and an appearance offset map that can be used to model newly appeared MS lesions.

New lesions detection and segmentation performances were compared to approaches scoring best in the MSSEG-2 challenge. The proposed CNN achieved highest lesion sensitivity (proportion of detected ground truth lesions) and F_1_-Score. Most automatic methods for new MS lesions segmentation tend to produce quite a lot of false positives. ANCR-Net was the only method capable of keeping the number of such false positives comparably low while still detecting 63.3% of the new lesions on average. Thus, our method is the one best suited to separate stable and progressing patients.

Segmentation performances overall were quite low, but even the medical experts achieved an average Dice score of only 0.573. Our method achieved the second-best Dice score of all automatic methods, with a value of 0.470. Evaluations on lesion level showed that correctly detected lesions are indeed well delineated, a fact that the overall Dice score fails to reflect. Whether the exact delineation of the new lesions is actually crucial for MS monitoring, or rather their number and size, should be further investigated. Here, our network could be a valuable tool as it estimated the true number of new lesions very well, with a mean deviation of only 1.3 lesions.

The modeled new lesions were shown to fit well with the intensity profile of the baseline images and were able to match the baseline to the follow-up image. Some modeled lesions exhibit an irregular intensity profile that might give new insights into the morphology of MS lesions. The intensity profile of the lesions can be analyzed independently of the surrounding MR images using our masked appearance offsets maps. Distracting or influencing factors of the original images can thus be eliminated. Extensions to multimodal network inputs would also allow analyzing different types of MS lesions. Sheng et al. for example differentiate between hypo-, iso- and hyperintense lesions on susceptibility-weighted imaging (Sheng et al., 2019). Such a distinction could easily be made automatically based on our modeled lesions.

Network training using random intensity transformations makes the method robust to appearance variations between time points, as they might e.g., be introduced by imaging artifacts (see also Section 3 in Supplementary material). Still, the challenge training data is limited to 40 cases with high quality and well pre-registered images, thus performance may degrade in less controlled settings. The training dataset should therefore be extended with more data that reflects the natural variability of images in clinical practice. For example, the images could be noisier, or they could have been taken with different scanners at each visit. Also, the current method is designed for monomodal data. Extensions to multimodal inputs could be achieved by training ANCR-Net for each modality separately and then combining the results for the different modalities. How the method can be extended to take advantage of the different modalities in a single CNN will be the subject of future research.

Overall, the automatic analysis of new MS lesions remains a very difficult task. Our network achieves good values for all metrics considered, performing comparable to state-of-the-art methods for new MS lesions detection and segmentation. It is the only method capable of reliably separating stable and progressing patients, which additionally allows estimating the real new lesion load. Beyond that, the generated appearance offset maps offer the possibility to investigate morphology and intensity profile patterns of newly developed MS lesions. Our method is thus an important step toward automating the analysis of new MS lesions and achieving the performance of medical experts.

## Data availability statement

Publicly available datasets were analyzed in this study. This data can be found here: https://shanoir.irisa.fr/shanoir-ng/challenge-request.

## Author contributions

JA, HU, JE, and TK: methodology. JA and JE: software. JA: validation and writing—original draft preparation. JA, HU, JE, TK, and HH: writing—review and editing. HH: supervision. All authors have read and agreed to the published version of the manuscript.

## Conflict of interest

The authors declare that the research was conducted in the absence of any commercial or financial relationships that could be construed as a potential conflict of interest.

## Publisher's note

All claims expressed in this article are solely those of the authors and do not necessarily represent those of their affiliated organizations, or those of the publisher, the editors and the reviewers. Any product that may be evaluated in this article, or claim that may be made by its manufacturer, is not guaranteed or endorsed by the publisher.

## Footnotes

^1^ https://github.com/Inria-Empenn/lesion-segmentation-challenge-miccai21/
