# Supplementary material for: Image registration and appearance adaptation in non-correspondent image regions for new MS lesions detection
Source: Front Neurosci. 2022 Sep 7;16:981523. doi: 10.3389/fnins.2022.981523 (PMC9490269; doi:10.3389/fnins.2022.981523)
Supplement: Supplementary file 1 [file Data_Sheet_1.pdf]

Supplementary Material

1 RESULTS PER PATIENT

In this section, new multiple sclerosis lesions detection and segmentation results are reported for each patient in the test data of MSSEG-2 challenge separately. In our observations, the segmentation of patient 12 appeared to be incorrect, which is why patient 12 was excluded from our evaluations. First, results are reported for all test patients with at least one new lesion in the manual ground truth in Tab. S1. Second, we report the number and volume of erroneously segmented lesions for patients without any new lesion in the manual ground truth in Tab. S2.

Patient ID	Nr.	FN	FP	TP_G	TP_A	F_1	$Sens_L$	PPV_L	Dice	HD	SD
01	15	6	2	9	9	0.692	0.600	0.818	0.663	40.635	0.002
02	2	1	4	1	1	0.286	0.500	0.200	0.432	68.387	16.862
05	1	0	1	1	1	0.667	1.000	0.500	0.646	52.772	0.095
06	2	0	0	2	2	1.000	1.000	1.000	0.773	1.676	0.006
08	4	0	1	4	4	0.889	1.000	0.800	0.531	21.119	0.008
11	2	0	0	2	2	1.000	1.000	1.000	0.577	3.319	0.000
17	10	5	2	5	5	0.588	0.500	0.714	0.358	51.655	2.472
22	2	2	5	0	0	0.000	0.000	0.000	0.000	82.241	26.131
23	16	3	6	13	13	0.743	0.813	0.684	0.649	41.289	0.010
25	1	0	4	1	1	0.333	1.000	0.200	0.449	38.828	4.683
31	1	1	0	0	0	0.000	0.000	0.000	0.000	-	-
33	1	0	0	1	1	1.000	1.000	1.000	0.880	1.623	0.007
36	10	2	3	8	8	0.762	0.800	0.727	0.588	32.552	0.493
40	13	4	1	9	9	0.783	0.692	0.900	0.579	36.006	0.054
44	5	0	1	5	5	0.909	1.000	0.833	0.620	49.842	0.013
46	12	4	1	8	8	0.762	0.667	0.889	0.655	44.472	0.004
53	3	0	1	3	3	0.857	1.000	0.750	0.455	20.584	0.023
56	3	1	1	2	2	0.667	0.667	0.667	0.160	54.710	31.886
60	25	10	1	15	15	0.732	0.600	0.938	0.893	48.506	0.664
62	39	17	8	22	22	0.638	0.564	0.733	0.482	45.111	0.573
66	45	15	5	30	32	0.753	0.667	0.865	0.574	29.997	0.005
67	1	1	1	0	0	0.000	0.000	0.000	0.000	68.932	66.913
75	1	0	1	1	1	0.667	1.000	0.500	0.827	70.590	0.007
78	1	1	0	0	0	0.000	0.000	0.000	0.000	-	-
80	2	0	0	2	2	1.000	1.000	1.000	0.699	1.381	0.035
81	5	1	8	4	4	0.471	0.800	0.333	0.446	71.578	11.635
82	2	1	1	1	1	0.500	0.500	0.500	0.779	48.393	6.915
85	1	1	4	0	0	0.000	0.000	0.000	0.000	98.302	42.024
86	1	1	4	0	0	0.000	0.000	0.000	0.000	58.728	34.767
87	1	0	0	1	1	1.000	1.000	1.000	0.820	2.396	0.048
97	4	3	1	1	1	0.333	0.250	0.500	0.032	50.300	16.195

Table S1. New MS lesions detection and segmentation results for progressing patients with at least one new lesion in the manual ground truth data. For each patient, the number of ground truth lesions, the number of false negative (FN) and false positive (FP) lesions, the number of ground truth lesions correctly identified (TP_G), and the number of segmented lesions overlapping with the ground truth (TP_A) are reported. For lesion detection, we state F_1-Score, lesion sensitivity ($Sens_L$) and lesion positive predictive value (PPV_L). Finally, for lesion segmentation we report Dice score, Hausdorff (HD) and surface distance (SD).

Patient ID	Nr.	Volume	Patient ID	Nr.	Volume	Patient ID	Nr.	Volume
03	0	0.000	42	0	0.000	71	0	0.000
04	1	7.750	45	0	0.000	72	0	0.000
07	0	0.000	50	0	0.000	73	0	0.000
09	1	35.000	54	0	0.000	76	0	0.000
10	0	0.000	55	0	0.000	79	0	0.000
14	0	0.000	58	0	0.000	92	0	0.000
28	0	0.000	59	0	0.000	93	1	14.355
34	0	0.000	63	0	0.000	98	0	0.000
38	0	0.000	64	0	0.000			
41	0	0.000	65	0	0.000			

Table S2. New MS lesions detection results for stable patients with no new lesion in the manual ground truth data. For each patient, the number and volume of erroneously detected lesions are reported.

2 MODELING OF NEW LESIONS

In the following, additional examples of the new multiple sclerosis lesions modelled by ANCR-Net are shown for patients 5, 23, 40, 44, 60, 62, 75 and 87 in Fig. S1 - S4. In each figure, we show baseline and follow-up MR images, as well as the subtraction image. Furthermore, the outputs of ANCR-Net are displayed: Masked appearance offset map, the spatially deformed and appearance adapted baseline image, appearance offset map, segmentation of new MS lesions and the subtraction image after registration and appearance adaptation.

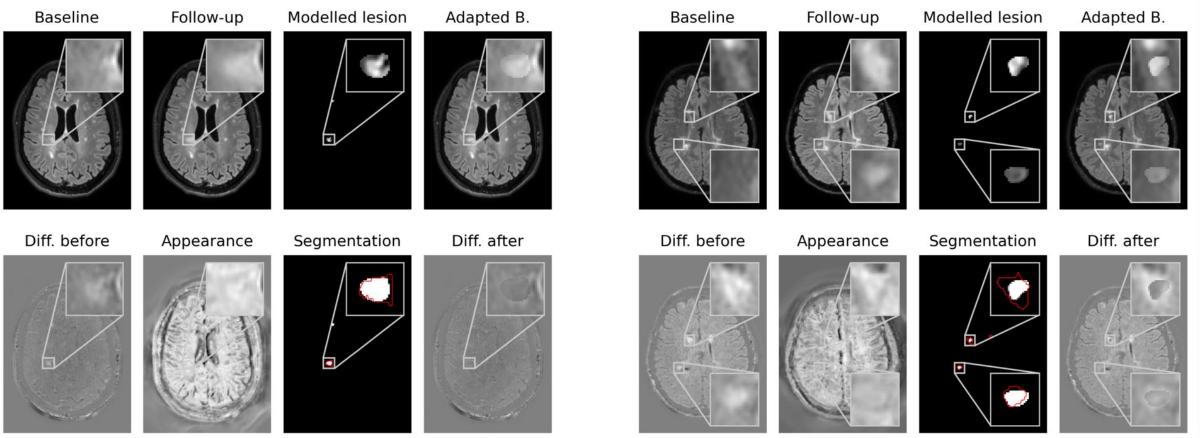

Figure S1. New MS lesions modeling results for patient 5 (slice 162) on the left and patient 23 (slice 155) on the right.

3 THE INFLUENCE OF APPEARANCE DIFFERENCES ON SEGMENTATION AND DETECTION PERFORMANCE

Finally, we want to analyze the robustness of our proposed method to variations in image appearance. Since ANCR-Net associates appearance changes with lesions, differences in overall appearance should not affect the performance of the network to a certain extent. We measure the robustness by artificially disturbing the input images and examining the performance of ANCR-Net for different degrees of perturbation. The artificially introduced disturbances comprise typical MR imaging artifacts (ghosting and bias fields), blurring, Gaussian noise and simulated reduced resolution during image acquisition

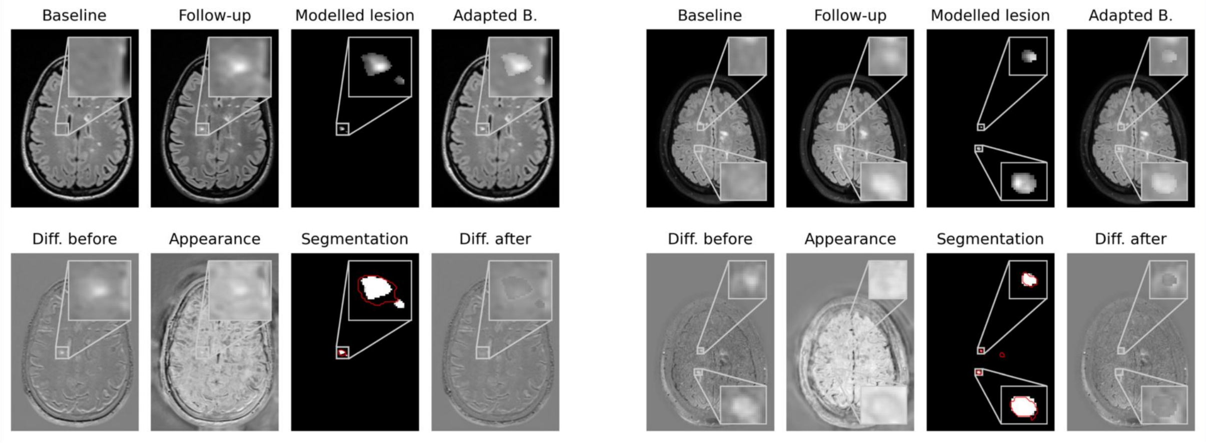

Figure S2. New MS lesions modeling results for patient 40 (slice 360) on the left and patient 44 (slice 161) on the right.

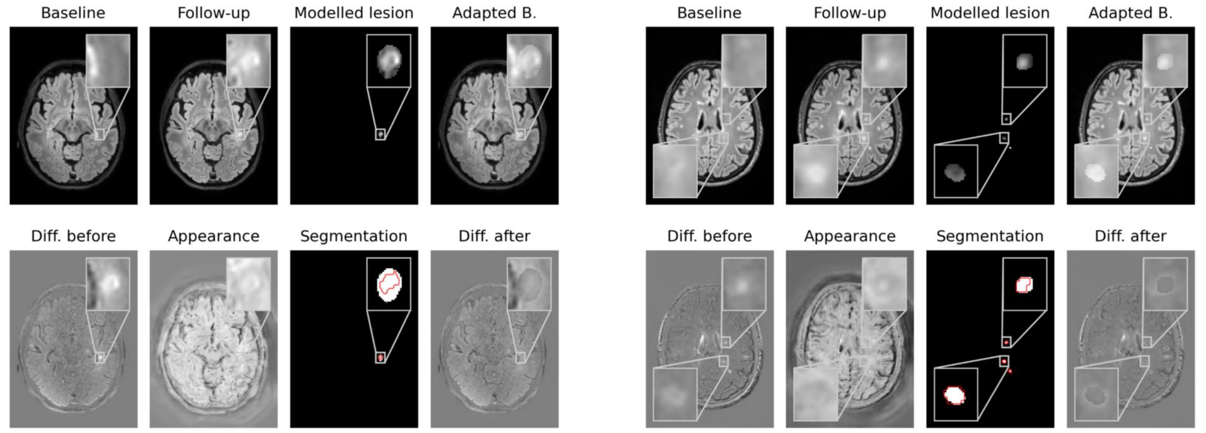

Figure S3. New MS lesions modeling results for patient 60 (slice 136) on the left and patient 62 (slice 187) on the right.

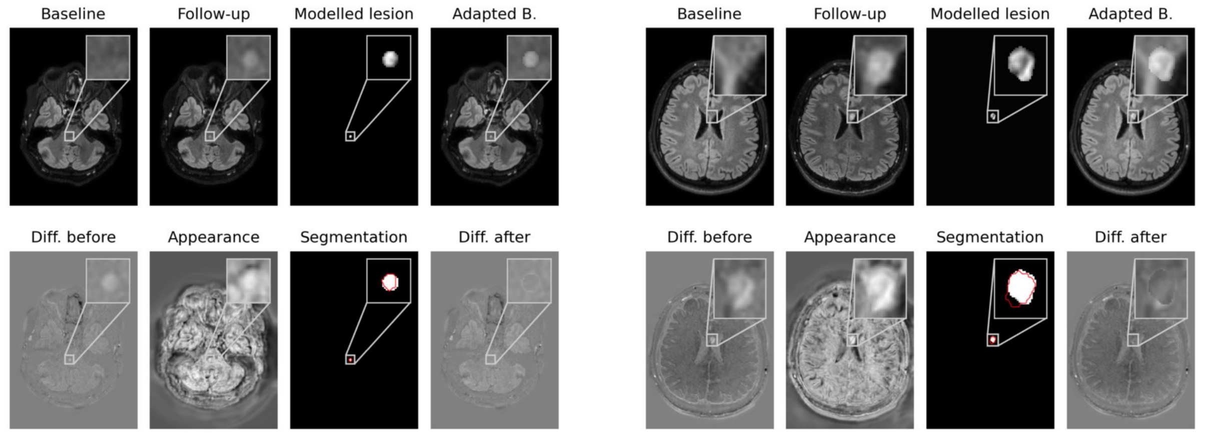

Figure S4. New MS lesions modeling results for patient 75 (slice 124) on the left and patient 87 (slice 138) on the right.

(anisotropic downsampling). For each of the introduced disturbances, we manually choose a maximum level, which we consider as the level that no longer allows to examine the images. We perform three experiments for each perturbation, disturbing either the baseline, the follow-up or both images before passing the images through our CNN. When disturbing both MR images, it is ensured that directional disturbances, such as ghosting or bias fields, are applied in different directions for baseline and follow-up. The performance of ANCR-Net for the different disturbance levels is shown in Fig. S5, along with sample images showing the introduced distortions. Displayed are the detection rates of stable and progressive patients, as well as the Dice score for progressive and the number of erroneously segmented lesions for stable patients.

Results for either disturbed baseline or follow-up show that our proposed method delivers stable detection rates for most disturbances, despite significant appearance differences between time points. Solely ghosting artifacts and heavy Gaussian noise lead to a greater drop in detection rates. This shows that our ANCR-Net may be used for new MS lesions detection in the presence of imaging artifacts and appearance differences between time points, as they might appear when different scanners are used for image acquisition. The segmentation accuracy decreases as the disturbance level increases, where perturbations in the baseline image tend to have less of a negative impact. Since the lesion borders of new lesions are only visible in the follow-up, this behavior is as expected. In contrast, disturbances of the baseline images are more likely to result in false positive lesions.

Additionally, it can be seen that the performance of ANCR-Net is especially robust against disturbances that have been covered during network training. The random intensity gradients applied during training are similar to the bias fields used in this experiment. Also, the dataset comprises images with substantially different resolutions, which is reflected by the anisotropically downsampled images. To further improve the network's robustness, additional augmentations may be used during training. For example, ghosting or other imaging artifacts have not been covered by our training procedure, but might be introduced to stabilize ANCR-Net for the natural variability of images found in clinical practice.

Overall, the segmentation performance of ANCR-Net suffers from substantial image disturbances, but detection rates are robust against a wide variety of imaging artifacts. Even in the presence of substantial overall appearance differences between time points, our proposed method is still able to separate stable and progressive patients.

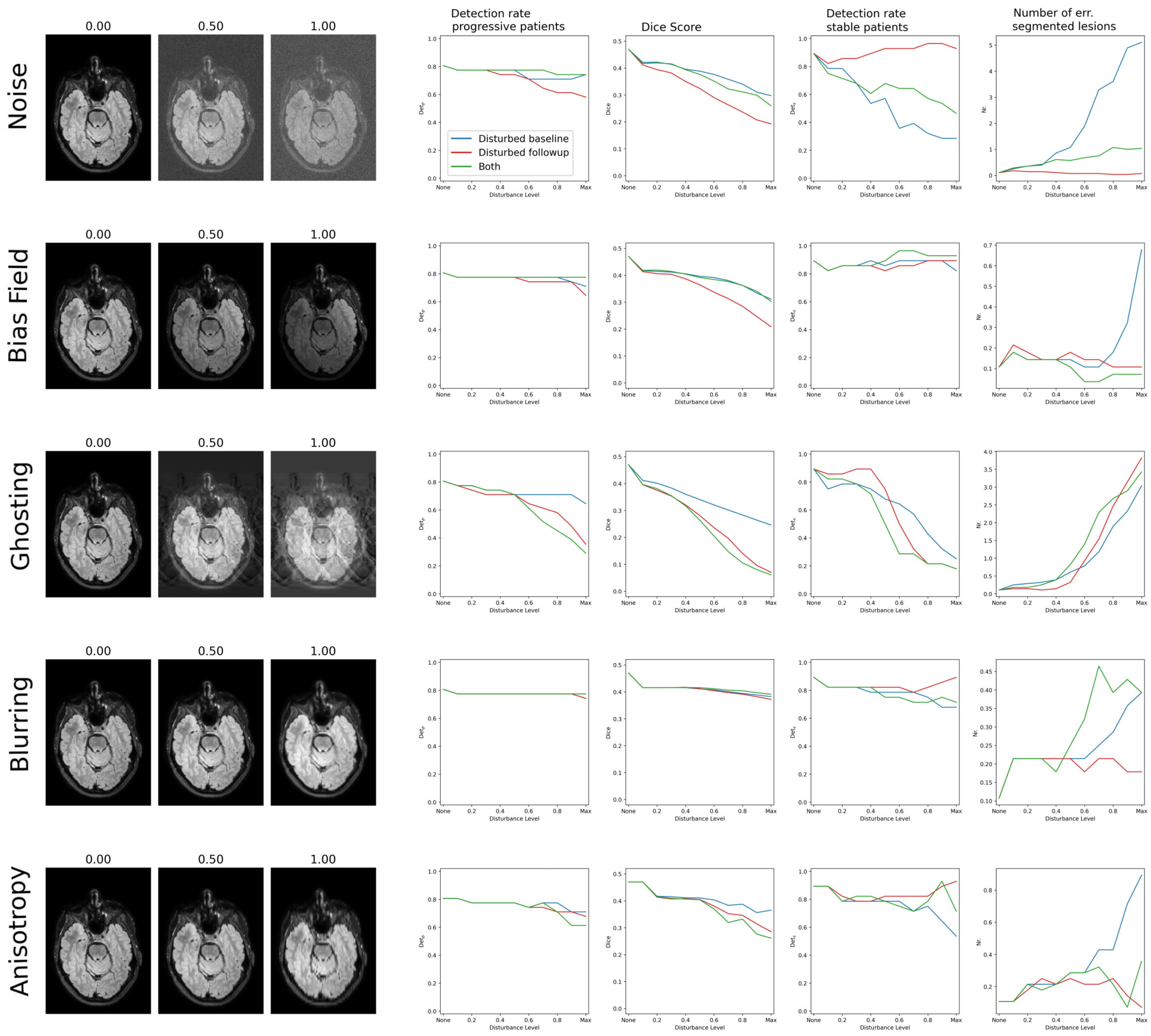

Figure S5. Influence of appearance differences and imaging artifacts on the performance of ANCR-Net. The input images are increasingly disturbed and passed through the network. New lesions detection and segmentation metrics are shown for the different disturbance levels for progressive and stable patients. Blue lines show results when only the baseline image is corrupted, red lines when only the follow-up image is corrupted, and green lines show results when both input images are corrupted. A disturbed input sample is shown on the left side for different disturbance levels.
